# High Throughput Screening of Antimicrobial Resistance Genes in Gram-Negative Seafood Bacteria

**DOI:** 10.3390/microorganisms10061225

**Published:** 2022-06-15

**Authors:** Sabine Delannoy, Corine Hoffer, Raphaëlle Youf, Emilie Dauvergne, Hattie E. Webb, Thomas Brauge, Mai-Lan Tran, Graziella Midelet, Sophie A. Granier, Marisa Haenni, Patrick Fach, Anne Brisabois

**Affiliations:** 1 COLiPATH Unit & Genomics Platform IdentyPath, Laboratory for Food Safety, ANSES, 94700 Maisons-Alfort, France; hoffercorine@gmail.com (C.H.); raphaelle.youf@univ-brest.fr (R.Y.); emilie.dauvergne@etud.u-picardie.fr (E.D.); mai-lan.tran@anses.fr (M.-L.T.); patrick.fach@anses.fr (P.F.); 2 Department of Animal and Food Sciences, International Center for Food Safety Excellence, Texas Tech University, Lubbock, TX 79409, USA; hwebb@cdc.gov; 3 Bacteriology and Parasitology of Fishery and Aquaculture Products Unit, Laboratory for Food Safety, ANSES, 62200 Boulogne-sur-Mer, France; thomas.brauge@anses.fr (T.B.); graziella.midelet@anses.fr (G.M.); 4 Antibiotics, Biocides, Residues and Resistance Unit, Fougères Laboratory, ANSES, 35306 Fougères, France; sophie.granier@anses.fr; 5 Antimicrobial Resistance and Bacterial Virulence Unit, Lyon Laboratory, Université de Lyon, ANSES, 69364 Lyon, France; marisa.haenni@anses.fr; 6 Strategy and Programs Department, ANSES, 94700 Maisons-Alfort, France; anne.brisabois@anses.fr

**Keywords:** antimicrobial resistance genes, mobile genetic elements, seafood bacteria, Gram-negative species, high throughput qPCR, micro-array

## Abstract

From a global view of antimicrobial resistance over different sectors, seafood and the marine environment are often considered as potential reservoirs of antimicrobial resistance genes (ARGs) and mobile genetic elements (MGEs); however, there are few studies and sparse results on this sector. This study aims to provide new data and insights regarding the content of resistance markers in various seafood samples and sources, and therefore the potential exposure to humans in a global One Health approach. An innovative high throughput qPCR screening was developed and validated in order to simultaneously investigate the presence of 41 ARGs and 33 MGEs including plasmid replicons, integrons, and insertion sequences in Gram-negative bacteria. Analysis of 268 seafood isolates from the bacterial microflora of cod (*n* = 24), shellfish (*n* = 66), flat fishes (*n* = 53), shrimp (*n* = 10), and horse mackerel (*n* = 115) show the occurrence of *sul-1*, *ant(3*″*)-Ia*, *aph(3*′*)-Ia*, *strA*, *strB*, *dfrA1*, *qnrA*, and *bla*_CTX-M-9_ genes in *Pseudomonas* spp., *Providencia* spp., *Klebsiella* spp., *Proteus* spp., and *Shewanella* spp. isolates and the presence of MGEs in all bacterial species investigated. We found that the occurrence of MGE may be associated with the seafood type and the environmental, farming, and harvest conditions. Moreover, even if MGE were detected in half of the seafood isolates investigated, association with ARG was only identified for twelve isolates. The results corroborate the hypothesis that the incidence of antimicrobial-resistant bacteria (ARB) and ARG decreases with increasing distance from potential sources of fecal contamination. This unique and original high throughput micro-array designed for the screening of ARG and MGE in Gram-negative bacteria could be easily implementable for monitoring antimicrobial resistance gene markers in diverse contexts.

## 1. Introduction

Antimicrobial resistance (AMR) is a global public health safety issue since antimicrobial-resistant bacteria (ARB) can reduce treatment options and efficiency, especially in Gram-negative bacterial infections [1]. AMR affects both humans and animals, therefore the dissemination of ARB through the food chain and the environment has become a major concern underlined by the World Health Organization [2].

Tackling AMR in animals, food, and humans in a One Health approach is a key priority for public health organizations worldwide as highlighted in the Global Action Plan on AMR from the tripartite Alliance [3]. Current AMR monitoring programs and risk assessment studies on food as a potential AMR transmission route focus on terrestrial food-producing animals; however, data on AMR in seafood are limited. Nevertheless, bacteria of terrestrial origin, including those conferring AMR, can reach the aquatic environments through runoff from land, faeces from wild animals and birds, or through sewage systems [4,5]. Furthermore, residues of antibiotics in rivers can favour the persistence of ARB, while the use of antimicrobials in aquaculture can directly contribute to AMR dissemination [6]. Furthermore, the use of other chemicals such as biocides, commonly used in seafood production and processing facilities, or antifouling agents (containing heavy metals) used on boat hulls, are known to co-select for AMR [7,8,9]. Thus, environmental ecosystems represent a potential reservoir of antimicrobial resistance genes (ARGs) and ARB that can spread to humans through direct contact with animals or consumption of food products. Conversely, anthropogenic activities are a source of ARGs in the environment and need to be investigated [10]. Beyond vertical transmission, ARGs can spread horizontally via mobile genetic elements (MGEs). The latter comprise insertion sequences, transposons, integrons, plasmids, and integrative and conjugative elements (ICEs), which allow the transfer of ARGs to other bacterial cells and, therefore, facilitate the spread of antimicrobial resistance between bacteria sharing the same ecological niche [11,12].

The marine ecosystem can be considered as a large reservoir of ARB and ARGs mostly acquired through faecal contamination of human and/or animal origin. Although global seafood production is growing each year, systematic surveillance of AMR in this sector is largely lacking or has only been at a pilot study level. One main reason for this gap is certainly the difficulties of investigating AMR phenotypes of marine bacteria, which often have specific growth requirements, and often lack standardised antimicrobial susceptibility testing methods and interpretation criteria. Consequently, molecular-based methods such as polymerase chain reaction (PCR) [13], whole genome sequencing (WGS) of isolated bacteria, or metagenomic approaches are favoured nowadays. Several high throughput qPCR methods for analysing hundreds of ARGs in a single experiment have recently been developed and their application has been powerful on environmental samples [14]. Nevertheless, most of them are based on the abundance of targeted genes in bacterial communities and disparities in data analysis have been highlighted [14]. WGS is also a powerful tool to detect ARGs and track potential transmission events [15], but it relies on a bacterial isolation step that can be challenging for marine bacteria. Although commonly used in developed countries for the surveillance of major zoonotic bacteria and foodborne investigations, WGS is still costly, which limits its routine use in a sector where no regulation exists. Further, next generation sequencing (NGS) has also opened the door to metagenomic studies, which are also expensive and complex in their analysis; nonetheless, these methods are of great interest when working with complex sample matrices.

The main objective of this study was to investigate ARGs and MGEs in seafood bacteria, in order to estimate the AMR burden conveyed by the marine sector. For this purpose, we developed and validated an original method, based on high throughput real-time PCR technology, to detect a selection of the most prevalent ARGs and some MGEs commonly found in Gram-negative bacteria using a dynamic microarray “AMR Array” with the Biomark system (Fluidigm). We then applied the developed AMR Array on a selection of Gram-negative bacteria isolated from marine sources.

## 2. Materials and Methods

### 2.1. Collection of Bacterial Strains Isolated from Seafood

**Figure 1:**
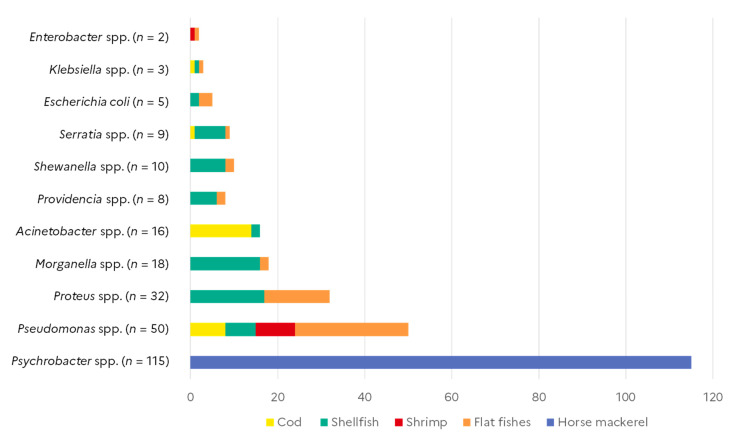
Bacterial isolates studied and their seafood sources.

A collection of 268 selected Gram-negative isolates recovered from commonly consumed seafood was selected from the Anses collection [16]. Bacterial strains were isolated from cod–*Gadus morhua* (*n* = 24), flat fishes (*n* = 53), pelagic fishes/horse mackerel–*Trachurus trachurus* (*n* = 115), shellfish (*n* = 66), and shrimp (*n* = 10). Strains were isolated from several organ samples of fresh seafood (skin and gills for fishes), except for horse mackerels which were collected during a sea cruise [17] and were frozen before the analysis [16]. Identification of the genus and species level, when possible, was performed using Maldi-TOF spectra or 16S rDNA sequencing [18]. In total, isolates belonged to eleven different genus or species, *Psychrobacter* spp. (*n* = 115), *Pseudomonas* spp. (*n* = 50), *Proteus* spp. (*n* = 32), *Morganella* spp. (*n* = 18), *Acinetobacter* spp. (*n* = 16), *Providencia* spp. (*n* = 8), *Shewanella* spp. (*n* = 10), *Seratia* spp. (*n* = 9), *Escherichia coli* (*n* = 5), *Klebsiella* spp. (*n* = 3), and *Enterobacter* spp. (*n* = 2) (Figure 1). The Gram-negative bacteria we chose were those recognised by WHO as clinically relevant in terms of critical antimicrobial resistance. The WHO list of pathogenic bacteria mainly includes Gram-negative bacteria belonging to several genus such as *Acinetobacter*, *Pseudomonas*, and various Enterobacteriaceae (including *Klebsiella*, *E. coli*, *Serratia*, and *Proteus*) [19]. In contrast, *Psychrobacter* isolates were recovered from a dedicated sampling from horse mackerel and considered as ubiquitous bacteria isolated from various food and environmental sources.

### 2.2. Selection of Antimicrobial Resistance Markers and Primers Design

A set of 74 ARGs and MGEs were selected based on their public health significance and the role they can play in the dissemination of AMR within Gram-negative bacteria. Among the high number of ARGs that have been described in the literature, we selected those that are the most prevalent or those encoding resistances to critically important antimicrobials for human medicine [19]. Among them, genes conferring resistances to beta-lactams (including cephalosporins and carbapenems), polymyxins, quinolones, aminoglycosides, phenicols, sulfonamides, trimethoprim, and tetracyclines were selected (Table 1). The MGE set included the three *intI* integron determinants [20], 16 insertion sequence markers, 13 plasmid replicon sequences belonging to the main incompatibility groups [21], and the Tn*2* transposon (Table 2).

Primers and Taqman™ probes targeting ARG and MGE markers were either recovered from the literature [22,23,24,25,26,27] or designed for this project (Table S1). For the design of primers and probes, all available sequences of the target region were collected from existing databases (NCBI, ResFinder (downloaded on 16 February 2018), ARG-ANNOT v3 (downloaded on 6 February 2018), CARD (downloaded on 1 February 2018)), and aligned for consensus sequences using the CLC Genomic Workbench (Qiagen, Hilden, Germany). When necessary, multiple sets of primers and probes were designed to encompass the described diversity of the target sequence. The designed primers and probes were all controlled in silico and further validated against a panel of control reference strains and field strains already described and well-known for carrying the ARG or MGE (Table S2).

### 2.3. High Throughput Real-Time PCR System

The Biomark real-time PCR system (Fluidigm, San Francisco, CA, USA) was used for high-throughput microfluidic real-time PCR (HT qPCR) amplification using 96.96 dynamics arrays. DNA extraction was performed using the InstaGene matrix (BioRad Laboratories, Marnes-La-Coquette, Paris, France) according to the manufacturer’s instruction on a single colony of pure culture bacteria isolated on Mueller-Hinton agar (BioRad) or TSYe agar (BioMérieux, Marcy-l’Étoile, France). Dynamic AMR array (Fluidigm) was performed according to the manufacturer’s recommendations using 6-carboxyfluorescein (FAM) and 6-carboxy-2′,4′,5′,7′,7′-hexachlorofluorescein succinimidyl ester (HEX)-labeled TaqMan™ probes and PerfeCTa qPCR ToughMix (Quanta). The thermal profile for the Biomark PCR was used as previously described [28]. PCR cycling comprised of 2 min at 50 °C, 10 min at 95 °C, followed by 40 cycles of 2-step amplification of 15 s at 95 °C, and 1 min at 60 °C. Data were acquired on the Biomark™ Real-Time PCR System and analysed using the Fluidigm Real-time PCR Analysis software to obtain crossing point (CP) values. Testing of the marine bacterial collection included positive controls for each qPCR tests, negative control, as well as a 16S qPCR test as loading control.

### 2.4. Positive Controls

A collection of positive controls was set up to validate the HT qPCR tests. This control collection included strains (or DNA extracts thereof) for which the targeted ARG or MGE had already been confirmed and/or characterised by WGS. When no strain was available, plasmids with insertion of the targeted ARG or MGE sequences were constructed (Genecust, France). The overall collection included 81 strains (or DNA extracts thereof), 57 of which had been fully sequenced, and 15 recombinant plasmids so that at least one positive control for each target was included (Table S2). Inclusivity of each primer and probe was validated with either control reference strains or surrogate recombinant plasmids. Exclusivity was also checked to ensure no cross-reaction or non-specific detection occurred among the panel of control strains (Table S3).

## 3. Results

### 3.1. Validation of the AMR Array Design for Antimicrobial Resistance Markers Detection

A total of 41 different TaqMan real-time PCR markers were designed, which targeted common ARGs found in Gram-negative bacteria (Table 1). Gene markers for the following β-lactamases were included: TEM, SHV, CTX-M-1, CTX-M-2, CTX-M-8, and CTX-M-9 groups, KPC-2/3, PER1, VIM, and NDM-1/2. Plasmid-encoded ampC β-lactamase genes belonging to the CMY family (*bla*_CMY-1/2/3_) were also targeted. Among the large family of aminoglycoside resistance genes, marker variants of the aac, ant, and aph families were included, along with *armA* methylase and streptomycin-resistance genes *strA* and *strB* markers (also named *aph(6)-Ia* and *aph(6)-Id*, respectively). In addition, three quinolone resistance markers targeting *qnr* genes (*qnrA*, *qnrB1*, *qnrS*), three sulfonamide resistance markers targeting sul genes (*sul1*, *sul2*, *sul3*), two tetracycline resistance markers (*tet*(A) and *tet*(B)), dihydrofolate reductase genes conferring resistance to trimethoprim (*dfrA1*, *dfrA12*, *dfrA17*), six markers of mobile colistin resistance genes (*mcr*-1/2/3/4/5 and *mcr*-9), and a marker for *floR* gene encoding phenicol resistance were also included in the AMR-array design. All adequate primers and probes were tested and validated with either control reference strains or surrogate recombinant plasmids (Table S2). As shown in Table S3 all the primers/probes sets tested exhibited sensitivity and specificity of 100%, except for those targeting *ant(3*″*)-Ia*, *aph(3*′*)-la*, *floR*, and *sul3* with sensitivity between 86 and 93%, and those targeting *aac(3)-IIa*, *ant(3*″*)-Ia*, *aph(3*′*)-la*, and *floR* with specificity between 89 and 98%.

In this study, we selected sixteen IS elements frequently associated with antimicrobial resistance determinants, and the three classes of integron for investigation. In addition, thirteen markers for the major plasmid incompatibility groups and transposons of the Tn*2* family were also investigated. When necessary, several sets of primers and probes were used to cover the target genetic diversity. In total, 47 primers and TaqMan™ probe sets targeted 33 markers of the mobilome of the studied strains. Table S1 presents all targeted MGE. Some targets, especially plasmid replicons, required multiple primers and probes due to high genetic diversity. The sensitivity and specificity of these primers and probe sets were determined with control reference strains (Table S2) and are reported in Table S3. The sensitivity for all targets but eight targets was 100%. For these eight targets (IncFIB, IncFII, IncHI1B, IncI-I1, IS*Kpn26*, IS*903*, *intI1*, and Tn*2*) the sensitivity ranged from 75% to 97%. Similarly, for all targets but four (IS*Sen4*, IS*Kpn26*, IS*26*, *intI1*), the specificity was 100%. The specificity was found to be between 88 and 98% for IS*Sen4*, IS*Kpn26*, and *intI1*. Only IS*26* produced a significant number of false positive with a specificity of 50%. The positive predictive value (Number of True positives/(Number of True positives + Number of False positives)) of this test is 84%, thus a positive signal for IS*26* still indicates a strong probability of presence of IS*26*.

### 3.2. Detection of Antimicrobial Resistance Genes in the Collection of Marine Bacteria

**Figure 2:**
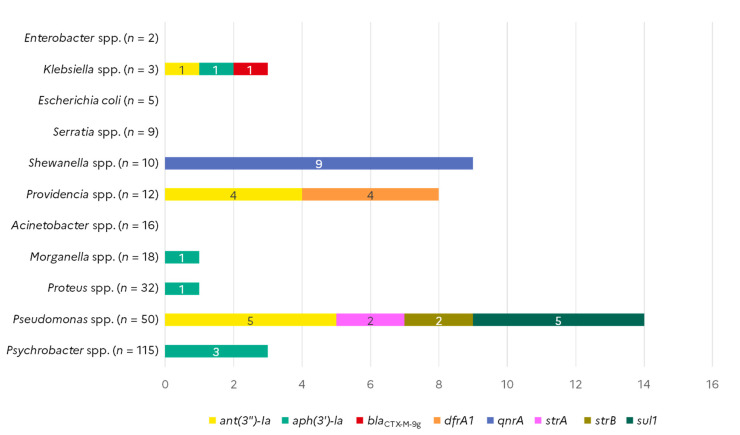
Occurrence of antimicrobial resistance genes in Gram-negative isolates from seafood (*N* = 268).

Among the 41 ARG markers investigated with the AMR array on the whole collection of strains, eight different markers detecting the *bla*_CTX-M-9_, *sul1*, *strA*, *strB*, *qnrA*, *dfrA1*, *aph*(*3*′*)-Ia*, and *ant*(*3*″*)-Ia* genes led to a positive signal (Figure 2). The sulfonamide resistance gene *sul1* was detected in five of the 50 *Pseudomonas* spp. isolates tested, which were all recovered from shrimp samples. The same five *Pseudomonas* spp. isolates also harboured the *ant*(*3*″*)-Ia* gene. Two other *Pseudomonas* spp. isolates from flat fishes were positive for *strA* and *strB* streptomycin resistance genes. The *ant*(*3*″*)-Ia* gene was also detected in four out of the eight *Providencia* spp. isolates collected from shellfish, and in one out of the three *Klebsiella oxytoca* isolate that came from flat fish. The same four *Providencia* co-harboured the dihydrofolate reductase *dfrA1* gene. The aminoglycoside phosphotransferase *aph*(*3*′*)-Ia* gene was detected in one *Klebsiella* spp. and one *Proteus vulgaris* isolate from different samples of shellfish, in one *Morganella morganii* isolate from a flat fish, and in three *Psychrobacter* spp. isolates collected from horse mackerel. The *aph*(*3*′*)-Ia* –positive *Klebsiella* spp. was the only isolate of this collection that presented an extended-spectrum beta-lactamase (ESBL) gene, namely a *bla*_CTX-M-9_ group gene. Out of the ten *Shewanella* spp. isolates, eight from shellfish (one of *Shewanella indica*, one of *Shewanella haliotis*, two of *Shewanella algae*, four of *Shewanella* spp.) and one from flat fish (*Shewanella haliotis*) harboured the plasmid-mediated quinolone-resistance *qnrA* gene. The single *Shewanella* spp. isolate that was negative for *qnrA* belonged to the *S. putrefaciens* species. Finally, no ARG was detected in *Acinetobacter* spp., *Serratia* spp., *E. coli*, and *Enterobacter* spp., from any of the sources (Figure 2). Furthermore, no ARG were found in any of the cod isolates.

### 3.3. Detection of Mobile Genetic Elements in the Collection of Marine Bacteria

**Figure 3:**
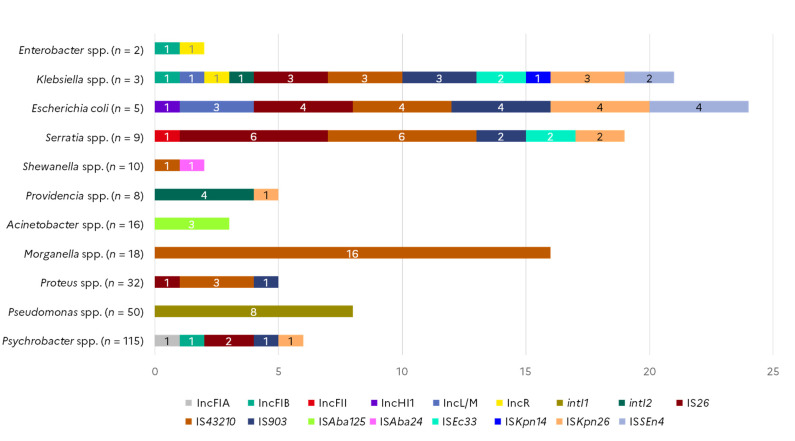
Occurrence of mobile genetic element markers in Gram-negative isolates from seafood (*N* = 268).

Among the thirteen plasmid incompatibility groups targeted, six were detected among the collection of marine bacteria (Figure 3). IncF plasmids can carry multiple types of replicons; in this study, we detected IncFII in one *Serratia quinivorans* isolate and IncFIA in one *Psychrobacter* spp. isolate from horse mackerel. The IncFIB replicon was identified in one *Klebsiella* spp. isolate collected from shellfish as well as in one *Enterobacter cloacae* and one *Psychrobacter* spp. isolate collected from shrimp. The IncHI1 replicon was detected in one *E. coli* isolate from shellfish. Three other *E. coli* and one *Klebsiella oxytoca* isolate from flat fish samples carried an IncL replicon. The same *Klebsiella oxytoca* isolate, as well as one *Enterobacter cloacae* isolate collected from flat fish, also harboured an IncR replicon. Presence of integrase genes from the three classes of integrons were investigated. Eight *Pseudomonas* spp. isolates, seven of which were isolated from shrimps and one from shellfish, carried *intI1*, whereas *intI2* was detected in four *Providencia* spp. from shellfish and one *Klebsiella oxytoca* isolated from a flat fish sample. None of the isolates carried an *intI3*.

Among the sixteen IS elements targeted in different species of the collection of marine strains, nine were detected in several bacteria species. The presence of IS*26* was probable in six *Serratia* spp., four *E. coli*, three *Klebsiella* spp., two *Psychrobacter* spp., and one *Proteus* spp. isolate collected from shellfish, flat fishes, cod, and horse mackerel. IS*903* was found in the same *Klebsiella* spp., *E. coli*, and *Serratia* isolates positive for IS*26*, as well as in one *Proteus* and one *Psychrobacter* spp. isolate. IS*4321* was detected in 16 out of the 18 *Morganella* spp. tested, in all three *Klebsiella* spp. isolates, in four out of the five *E. coli* tested, in three *Proteus* spp. isolates, in seven *Serratia* spp., and one *Shewanella* spp. isolate. IS*Aba24* was detected in one *Shewanella* spp. while IS*Aba125* was found in three *Acinetobacter* spp. isolates. IS*Ec33* was detected in two *Klebsiella* spp. and two *Serratia* spp. isolates, IS*SEn4* marker was found in four *E. coli* and two *Klebsiella* spp. isolates. IS*Kpn14* was identified in one *Klebsiella* spp., while IS*Kpn26* was detected in all three *Klebsiella* spp., in four *E. coli*, two *Serratia* spp., and one *Providencia* spp. isolate.

### 3.4. Combined Detection of ARGs and MGEs

**Figure 4:**
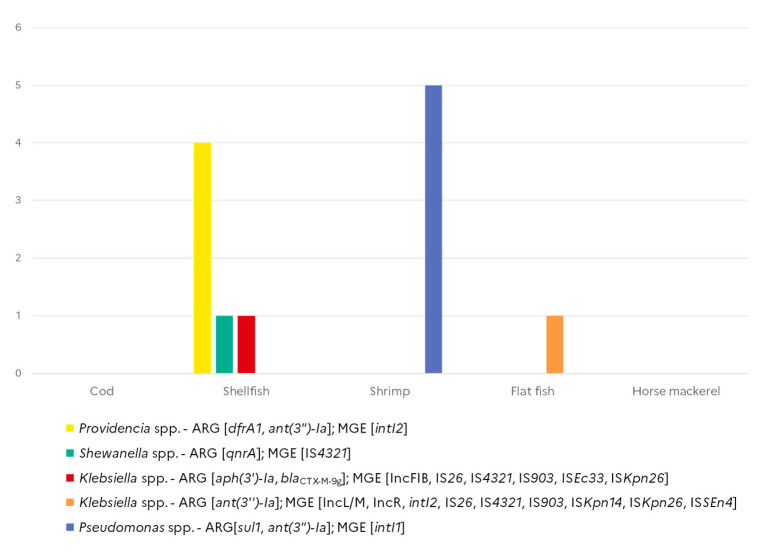
Co-occurrence of antimicrobial resistance genes and mobile genetic element markers in Gram-negative isolates from seafood (*N* = 268).

Out of the 268 strains tested, 76 (28.4%) gave a positive signal either for ARG (*n* = 27) or for MGE markers (*n* = 61), but only 12 carried both ARGs and MGEs (Figure 4). Among these 12 isolates, five *Pseudomonas* spp. isolated from shrimps harboured *sul1* and *ant(3*″*)-Ia* genes associated with *intI1*, while four *Providencia* spp. isolates collected from shellfish harboured the *ant(3*″*)-Ia* and *drfA1* genes together with *intI2*. Two *Klebsiella*, one isolated from shellfish and one *K. oxytoca* isolated from flat fish, simultaneously harboured ARGs as well as multiple IS elements and at least one plasmid marker. One of the *qnrA*-positive *Shewanella* spp. isolate was also found positive for IS*43210*.

## 4. Discussion

Antimicrobial resistance is a major public health issue and concerns not only human and veterinary sectors but also the overall environmental and food sectors. Therefore, detection and identification of ARG content in food and food-producing environments is complementary to the data available for human and animals sectors for a One Health approach as highlighted in in a recent EFSA scientific opinion [5]. Although AMR data on key pathogens exist for some seafood items, AMR surveillance has not been implemented as it has for the zoonosis monitoring plans in terrestrial food animals. In the 2017 report “A European One Health Action Plan against Antimicrobial resistance”, the European Commission calls for efforts to close knowledge gaps regarding the contribution of the environment to the AMR in humans and there is a need to determine occurrence and temporal trends of AMR in the environment including the marine sector [29].

Only a few studies have looked for the presence of ARB and ARGs in post-harvested fish and shellfish, but these have detected priority pathogens or fish pathogens and other commensal or opportunistic marine and freshwater bacteria [30]. A study of wild fish in Algerian Mediterranean reported 21.3% of ESBL-producing carriage in *E. coli*, *K. pneumoniae*, *Enterobacter clocae*, *Morganella morganii*, *Citrobacter freundii*, and *Proteus vulgaris* presumably contaminated with untreated human sewage [31]. A study of wild freshwater fish reported ESBL-*E. coli* in 0–85% of sample depending on the geographical site of sampling and human faecal pollution [32]. Few studies have reported ESBL-producing *Enterobacteriaceae* in shellfish production and retail [33,34]. In all, available data on AMR in marine and seafood bacteria are scarce and diverse according to the source of samples, targeted bacteria, and methodologies used for AMR or ARG detection. Although global seafood production is growing each year, systematic surveillance of AMR in this sector is largely lacking or is only at a pilot study level. One main reason for this gap is certainly the difficulties of investigating the AMR phenotypes of marine bacteria, which often have specific growth requirements, in the absence of any standardised methods or interpretation criteria.

In this study, we focused on selected ARGs that are of highest priority for public health concern, frequently plasmid-mediated and associated with various mobile genetic elements, including integrons, transposons, or insertion sequences. Investigation of MGEs is complementary to the ARG detection and brings insights on the potential capacity of ARGs to disseminate [35]. High throughput qPCR is a fast and convenient method for the simultaneous investigation of a large number of genes or genetic markers, and can advantageously be used to screen hundreds of ARGs or MGEs. The AMR-Array developed here offers a powerful tool to explore ARG and MGE content of seafood bacteria and therefore the potential exposure to human through seafood consumption. Here, we explored the role of seafood-associated bacteria as a potential reservoir and route of diffusion of antimicrobial resistant bacteria isolated from four different types and geographical sources of seafood, shrimp and shellfish, which are farmed products, whereas cod, horse-mackerel, and flat fishes are wild fishes.

Our results revealed the presence of several insertion sequences, plasmids, and class 1 and class 2 integrons in fifty-five isolates. All of these targeted MGEs have the ability to contain gene cassettes encoding resistance to several antimicrobials. They are known to carry integrons, which can contain genes for site-specific recombination and are capable of capturing and mobilising gene cassettes. In this study, five *Pseudomonas* spp. isolates from shrimp carried a class-1 integron and *sul1* and *ant(3*″*)-Ia* genes encoding resistance to sulfonamides and aminoglycosides. Class-1 integrons harbouring such gene cassettes are common [36,37]; and were previously identified in *Enterobacter cloacae*, *Aeromonas hydrophila*, *Klebsiella oxytoca*, and *Citrobacter freundii* in seafood from Japan [38], fish farms in Egypt [39], *Aeromonas* spp. isolated from rainbow trout in Australia [40], fresh fish in Mexico [41], *E. coli* from commercial fish and seafood in Korea [42], as well as *Salmonella enterica* from imported seafood in the United States [43]. None of these studies identified any class-2 integrons, whereas our study identified four *Providencia* spp. isolates from shellfish harbouring class-2 integron and *ant(3*″*)-Ia* and *drfA1* genes and one *Klebsiella oxytoca* strain isolated from flat fish carrying a class-2 integron, several insertion sequences (IS*26*, IS*903*, IS*4321*, IS*Kpn14*, IS*Kpn26*, IS*SEn4*), plasmid replicons (IncL/M and IncR), and the *ant*(*3*″*)-Ia* gene. Class-2 integrons have previously been found in *E. coli* isolates from samples of aquaculture water [44]. The presence of class-2 integrons with *dfrA1*- *ant(3*″*)-Ia* cassette arrays is common in the *Proteae* tribe [45]. Nevertheless, our study focused only on the three main classes of integrons whereas a recent publication mentioned the large diversity of the environmental pool of integrons and their role in the bacterial adaptation in response to environmental pressure [46].

IS*26* and IS*903* are frequently detected in clinical ESBL-producing *E. coli* and *Klebsiella pneumoniae* [47]. Interestingly, such IS were identified in the tested *Klebsiella* spp., *E. coli*, and *Serratia* spp. isolates; moreover, a CTX-M-9 group encoding gene was detected in one *Klebsiella* sp. isolated from shellfish. Such results suggest that seafood isolates could act as a reservoir of these IS associated with such ESBL genes. Furthermore, other genetic determinants of concern, such as ARGs not included in the microarray, heavy metals resistance, or other functions could be hosted. Thus, acquisition of ARGs on plasmids containing IS could occur further according to the ecological niches of the isolates, environmental factors, and selection pressures. Insertion sequence IS*4321* was detected in the same species of seafood isolates and in *Proteus* spp. and *Morganella* spp. isolates from shellfish. While no ARGs were detected in the isolates of the last two genus in this study, clinical *Proteus* and *Morganella* isolates often carry ARG cassettes and the TnpA transposase for IS*4321* [48,49]. However, none of the IS included in the microarray were found in the single *Proteus vulgaris* isolate carrying the *aph(3*′*)-Ia* gene. While it could be carried by another IS we did not target, it could also be carried by a non-mobile chromosomal locus.

Among the large variety of transferable plasmids described in *Enterobacteriaceae* [50], we targeted those that are known to be associated with ARGs. IncF, IncH, IncL/M conjugative plasmids, and IncR plasmid families were detected in only eleven isolates. IncR plasmids have been previously described in S*almonella* spp. and *Klebsiella* spp. [51]*;* furthermore, IncR have been reported carrying ARGs mediating resistance to five different classes of antibiotics in clinical *Enterobacterales* from different geographical regions [52,53]. Moreover, the pool of resistance genes carried by IncR plasmids may spread to transferable plasmids through transposition or plasmid recombination events [54]. Here, we detected two isolates carrying an IncR plasmid replicon: one *Enterobacter cloacae* strain from flat fishes, and one *K. oxytoca* from the same source that also carried an IncL/M replicon, several IS, and *intI2*. The review of Rozwandowicz reported IncHI1B frequently in MDR *K. pneumoniae* [55]. Our study identified one IncHI1B *E. coli* strain from shellfish. In addition, both plasmid replicons and ARG were only detected together in two isolates of *Klebsiella* spp., one *K. oxytoca* isolated from flat fish carrying *ant(3*″*)-Ia* gene, several IS and IncL/M as well IncR., other one isolated from shellfish carrying *aph(3*′*)-Ia*, *bla*_CTXM-9_ group genes, IncFIB, and several IS.

Finally, of the plasmid replicons, insertion sequences, and integrons targeted, we observed the presence of mobile genetic elements in 80%, 51%, 11%, 5%, and 4% of samples from shrimps, shellfish, flat fishes, horse mackerel, and cod, respectively. These results are consistent with the ecological niches of seafood samples. Indeed, cod and horse mackerel are deep sea wild fishes whose environment is rarely contaminated by anthropogenic sources and sewage. On the contrary, flat fishes (for example *Solea solea*, *Pleuronectes platesa*, *Limanda limanda*) belong to benthic species that preferentially live in shallow water near sandstone at the limit of fresh water, which are at much higher risk of being contaminated by *Enterobacteriaceae* (*E. coli*, *Klebsiella* spp., *Morganella morganii*, *Enterobacter cloacae*).

The presence of the *qnrA* gene was detected in 9 out of the 10 *Shewanella* spp. isolates tested. This gene has been described as naturally present in the chromosome of *Shewanella algae* and may have served as progenitor of *qnrA* acquisition in *Enterobacteriaceae* [56,57]. Our *Shewanella* isolates included two species other than *Shewanella algae* (*Shewanella haliotis*, *Shewanella indica)*, as well as four *Shewanella* isolates for which it was not possible to determine the species. These species could, like *Shewanella algae*, naturally harbour a *qnrA* or *qnrA*-like gene in their genomes detected by our device. Alternatively, they could also have received the *qnrA* gene mobilised long time ago from *Shewanella algae*, and circulating on many mobile genetic elements; this event could be of more public health concern than the prior one. We should highlight that we did not detect any of the MGE on our array in these isolates besides an IS*43210* in a single *Shewanella haliotis* isolate. This fact is much more in favour of an intrinsic resistance gene displayed by most of *Shewanella* spp. *Shewanella putrifaciens* is found mainly in marine environment, and contrary to other *Shewanella* species, no quinolone-mediated resistance *qnrA* genes was detected in our array for the *S. putrefaciens* isolate. The absence of the *qnrA* gene in *Shewanella putrefaciens* has already been described [56]. Interestingly, one single *Klebsiella* spp. strain recovered from shellfish carried a *bla*_CTXM-9_ group gene out of the *bla*_CTX-M_ genes of groups 1, 2, 8, and 9 tested, which are usually highly reported worldwide in clinical *E.coli* and *Klebsiella* spp. [58]. Such *bla*_CTX-M-9_ group genes have already been described in clinical *Klebsiella* spp., mostly in Asia, in line with the geographical origin of shellfish investigated here [58]. Nevertheless, only sparse data on *bla*_CTX-M_ genes in seafood bacteria are available.

The streptomycin resistance genes *strA-strB* were identified in a *Pseudomonas* isolate. The occurrence and expression of these genes has been already studied for *Pseudomonas syringae*, suggesting the importance of these bacteria as a potential reservoir of antibiotic resistance in the environment [59]. As MGEs have not been found in the *strA-strB* positive *Pseudomonas* isolate and in the *aph*(*3*′*)-Ia*-positive *Morganella morganii* isolate, these could be either chromosomally encoded genes or might have been mobilised by other MGE not included in this array. Only WGS and in silico analysis might be able to answer this question. Approximately half of the shellfish samples carried MGEs. These samples were composed of oysters, mussels, and scallops harvested on the coastline. Therefore, they are most likely under selective environmental pressure from sewage and other anthropogenic sources which can explain the frequent occurrence of positive MGE strains. Nevertheless, out of the fifty-five MGE-positive isolates, only six harboured ARGs: four *Providencia* spp. isolates positive for *ant(3*″*)-Ia* and *drfA1* that also carried an *intI2* integron, one *Klebsiella* spp. isolate positive for *aph(3*′*)-Ia* and *bla*_CTX-M-9_ group genes that also carried a IncFIB plasmid replicon and several IS elements, and one *Shewanella haliotis* isolate positive for *qnrA* and IS*4321*. Seven of the ten imported shrimp samples carried *intI1* and another one carried an IncFIB plasmid replicon.

We noticed the absence of ARGs detection in *Acinetobacter*, *E. coli*, and *Enterobacter* *cloacae* which usually carry ARGs, especially in clinical or animal isolates. Although we cannot preclude the presence of other, non-tested, rare ARGs, the absence of ARG in such marine strains in our study could be explained by a lack of antibiotic pressure in the marine niches even if other environmental factors might select for resistance. However, only few data are available to corroborate the hypothesis as most studies have been conducted on wastewater effluents.

## 5. Conclusions

Although it has been hypothesised that marine environments and seafood serve as a reservoir for ARGs and MGEs, only a few studies have been conducted on the bacterial flora of seafood to assess the prevalence of ARGs and MGEs. Here, we aimed to investigate some of the most prevalent ARGs and MGEs found in Gram-negative bacteria in seafood samples from different sources. We hypothesised that the occurrence of ARGs and MGEs could be associated with the nature of seafood and environmental, farming, and harvesting factors. Only 12 (4.5%) out of the 268 isolates displayed ARG-MGE associated patterns, which does not support the hypothesis of ARG transmission via horizontal transfer and the high level of human exposure directly or through seafood consumption. Nevertheless, MGE-positive isolates in seafood could act as reservoir for the acquisition of gene cassettes further along the food chain, i.e., during the transformation, distribution, or consumption of the seafood products, up to the clinical bacteria species recovered in human samples. These conclusions are in line with a recent report reviewing data on the contamination of aquatic and terrestrial environments in France for ARB and ARGs [4]. Our results corroborate the hypothesis that the proportions of ARB and ARGs decrease with increasing distance from the sources of contamination. The innovative AMR microarray designed in this study is a powerful high throughput tool, robust and easy to implement, for monitoring of some relevant ARGs and MGEs in any indicator selected bacteria from any specific ecosystem. It could also be a powerful tool for investigation of ARG and MGE content in complex DNA samples from various sources. This AMR microarray is easily adaptable and new relevant genetic markers can be implemented to accommodate the emergence of new ARGs.

## Figures and Tables

**Table 1:** Antimicrobial resistance genes (ARGs) selected for the AMR–Array design.

Antimicrobial Class	AMR Gene Familly	AMR Genes
Aminoglycosides	Aminoglycoside acetyltransferase (AAC)	*aac(6*′*)-Ib*, *aac(6*′*)-Ib-cr*, *aac(3)-IIa*, *aac(3)-IV*
	Aminoglycoside nucleotidyltransferase (ANT)	*ant(2*″*)-Ia*, *ant(3*″*)-Ia*
	Aminoglycoside phosphotransferase (APH)	*aph(3*′*)-Ia*, *strA (aph(3*″*)-Ib)*, *strB (aph(6)-Id)*
	16S rRNA methyltransferase	*armA*
β-lactams	(Extended-spectrum) β-lactamase	*bla*_TEM_
		*bla*_CTX-M group 1/2/8/9_
		*bla*_PER1_
		*bla*_SHV_
	Carbapenemase	*bla*_KPC-2/3_
		*bla*_VIM_
		*bla*_NDM-1/2_
	Other β-lactamase	*bla*_CMY-1/2/3_
Phenicols	Chloramphenicol exporter	*floR*
Fluoroquinolones	Quinolone resistance protein	*qnrA*, *qnrB1*, *qnrS*
Polymyxins	Phosphoethanolamine transferase	*mcr*-1/2/3/4/5/9
Folate pathway antagonists	Sulfonamide resistant dihydro-pteroate synthase Dihydrofolate reductase	*sul1/2/3* *dfrA1/12/17*
Tetracyclines	Tetracycline efflux pump	*tetA/B*

**Table 2:** Mobile genetic elements (MGEs) selected for the AMR–Array design.

MGE Family	MGE Name	AMR Genes Previously Reported with MGE
Plasmid incompatibility replicons	IncA/C, IncFIA, IncFIB, IncFII, IncX4, IncHI1, IncHI2, IncL/M, IncN, IncQ, IncR, Incl, IncK/B/O/Z,	*bla*_CMY_, *bla*_CTX-M_, *bla*_NDM_, *bla*_SHV_, *bla*_TEM_, *bla*_VIM_, *aac*/*aph*/*ant*, *armA*, *dfrA*, *qnr*, *floR*, *sul1/2/3*, *tetA/B*, *mcr1*
IS	IS*26*	*bla*_OXA-1_, *bla*_NDM_, *bla*_SHV-2_, *bla*_KPC_, *qnrB*, *mcr*-1
	IS*903*	disruption of *mgrB* (colistin efflux), *bla*_CTX-M_
	IS*4321*	*bla*_IMP_
	IS*6100*	*bla*_TEM_, *mphA*
	IS*Aba24/125*	*armA*, *strA/B*, *tetB*, *bla*_NDM_
	IS*Kpn14/26*	disruption of *mgrB* (colistin efflux), *mcr*-1
	IS*Kpn19/27*	*bla*_KPC-2_
	IS*Apl1*	*mcr*-1
	IS*Ec33*	*bla*_KPC_, *bla*_NDM_
	IS*Ecp1*	*bla*_CTX-M_
	IS*SEn4*	*bla*_NDM-1_
ISCR	IS*CR1*	*bla*_CMY_, *bla*_PER_, *bla*_CTX-M_, *qnrA*, *qnrVC*
	IS*CR3/14/27*	*bla*_VIM_, *bla*_NDM_
	Tn*2*	*bla*_TEM_
Integrons	*intI1*	aminoglycosides, β-lactams, phenicols, and macrolides
	*intI2*	*dfr1*
	*intI3*	*bla*_IMP-1_, *aacA4*, *bla*_GES_

## Supplementary Materials

The following are available online at https://www.mdpi.com/article/10.3390/microorganisms10061225/s1. Table S1: Primers and probes targeting Antimicrobial Resistance genes (ARG) and Mobile genetic elements (MGE) (see Refs. [22,23,24,25,26,27]). Table S2: Controls used to validate the assays. Table S3: Sensitivity and specificity of the assays used in this study.
