# Supplementary material for: High Throughput Screening of Antimicrobial Resistance Genes in Gram-Negative Seafood Bacteria
Source: Microorganisms. 2022 Jun 15;10(6):1225. doi: 10.3390/microorganisms10061225 (PMC9230514; doi:10.3390/microorganisms10061225)
Supplement: Supplementary file 1 [file microorganisms-10-01225-s001.zip › Table S1.pdf]

Table S1. Primers and probes targeting antimicrobial resistance genes (ARGs) and mobile genetic elements (MGEs).

Target	Primers and probes sequences	Reference
aac(6′)-Ib	F-AACTTGCGAGCGATCCGA<br>R-TGGCGTGTTTGAACCATGTAC<br>P-TACCTTGCTTCTCAAACCCCGCTTTCTC	[22]
aac(6′)-Ib-cr	F-AACTTGCGAGCGATCCGA<br>R-TGGCGTGTTTGAACCATGTAC<br>P-MGB-TACGGTACCTTGCCTCT	[22]
aac(3)-IIa	F-AGGACATCGTGACGTTCGG<br>R-AACCTGAAGGCTCGCAAGAG<br>P-CGGAGTGGTTCCGAAATGCTTCTCAAG	[22]
aac(3)-IV	F-ATCGGTCAGCTTCTCAACCT<br>R-TCGTCCAGACCTGACCAC<br>P-TCCACAGCTCCTTCCGTAGCGTCC	[22]
armA	F-GAAAGAGTCGCAACATTAAATGACTT<br>R-GATTGAAGCCACAACCAAAATCT<br>P-TCAAACATGTCTCATCTATT	[23]
ant(2")-Ia	F-CGGCGAGCTCGAGGC<br>R-ATTTCATACGCTTCGTCTGCC<br>P-TCATGGAGGAGTTGGACTATGGATTCTTAGCG	[22]
ant(3")-Ia	F-ACGTTGTCCCGCATTTGG<br>R-TCCTTCGGCGCGATTTT<br>P-MGB-ACAGCGCAGTAACC	[22]
aph(3′)-Ia	F-ACCGAGGCAGTTCCATAGGA<br>R-ACCTTATTTTTGACGAGGGGAAA<br>P-ATCCTGGTATCGGTCTGCGATTCCGA	[22]
strA	F-TCAATCCCGACTTCTTACCGG<br>R-CACCATGGCAAACAACCATATC<br>P-MGB-TGGCTCGTGTCGAAC	[22]
strB	F-ATCGCTTTGCAGCTTTGTTTC<br>R-TCCGAGGCATTGCTCATCATT<br>P-CGTCCACGCGGCGATTATAGCC	[22]
blaTEM	F-GGTATTATCCCGTRTTGACGC<br>R-GTCCTCCGATCGTTGTCAGAA<br>P-ACWCACCAGTCACAGAAAAGCATCTTMCG	This study
blaCTX-M-1group	F-AATCTGACGCTGGGTAAAGCR<br>R-CGTTGGTGGTRCCATAGYCAC<br>P-AGTCCWGCCTGAATRCTCGCTGCA	This study
blaCTX-M-2group	F-CGACGCTACCCCTGCTATT<br>R-CATCACCTTACTGGTACTGCA<br>P-AACGCCAAGCCGACCTCCCGAACTT	This study
blaCTX-M-8group	F-TAAGCGGATGATGCTAATGACAA<br>R-AACGTCGTTCGCCTGCGCA<br>P-CACTCCCCAGCAACAGCGAAATACAG	This study
blaCTX-M-9group	F-CGTGGCTCAAAGGCAATACGA<br>R-ATAGGTCACCAGAACCAGCG<br>P-ACCGCAATATCATTGGTGGTGCCGTAG	This study
blaKPC2/3	F-AGCGGCAGCAGTTTGTTGAT<br>R-ACGGCCAACACAATAGGT	[22]

	P-CAGTCGGAGACAAAACCGGAACCTGC	
blaNDM1/2	F-CGCAACACAGCCTGACTTT<br>R-CAGCCACCAAAAGCGATGTC<br>P-CCAGCAACCGCGCCCAACTTTGG	[22]
blaPER1	F-GGCTAAGGTTTTACAGAATACCT<br>R-GTCCACCAACCAGTTCAAATAAC<br>P-CGTATCAGGGAGACSAGTTTAGTGTTCC	This study
blaVIM	F1-GAGATTCCCACGCACTCTCTAGA<br>F2-GAGATTCCCACGCATTCTCTAGA<br>R-AATGCGCAGCACCAGGATAG<br>P-ACGCAGTGCGCTTCGGTCCAGT	[24]
blaSHV	F-TGACRAACAGCTGGAGCGAA<br>R-GCTGTTATCGCTCAYGGTAATG<br>P-TCCACTATMGCCAGCAGSATCTGG	This study
blaCMY-1	F-TGCTCAAGGATGGCAAGGC<br>R-TCCCTTGACCACCGCATAG<br>P-AGGGTCGCAGTCAGGGTCTTGCTCA	This study
blaCMY-2	F-AACCCTCAGGAATGAGTTACG<br>R-CAAGTTGTCCCGGAGAAACG<br>P-CAATGACCAGACGCGTCCTGCAACC	This study
blaCMY-3	F-GCTAACTCCAGYATTGGTCTG<br>R-CCAGGTATGCGCCAGTTTTAA<br>P-GACGCGTYTGGTCATYGCCTCTTC	This study
floR	F-AGATCGGATTCAGCTTTGCC<br>R-CAAAGGACTTCGCGAAACG<br>P-CGCCACTGTCGCGCTTGTAATGATC	[22]
qnrA	F-AGGATTGCAGTTTCATTGAAAGC<br>R-TGAACTCTATGCCAAAGCAGTTG<br>P-TATGCCGATCTGCGCGA	[25]
qnrB1	F-GTAGCGCATATATCACGAATACCAATC<br>R-ATCTGAACCACTGAACGTCGC<br>P-AAGTCGTGTTGGAAAAGTGTGAGCTGTGG	[22]
qnrS	F-CGACGWGCTAACTTGCGTGA<br>R-GGCATTGTTGGAAACTTGCA<br>P-TCATTGAACAGGGTGATATCGAAGGCTGC	[22]
mcr-1	F-TGGCGTTCAGCAGTCATTAT<br>R-AGCTTACCCACCGAGTAGAT<br>P-AGTTTCTTTCGCGTGCATAAGCCG	[26]
mcr-2	F-TTGTCGTGCTGTTATCCTATCG<br>R-CCGTGCCATAAGTATCGGTAAA<br>P-ACTGATTATGGGTGCGGTGACGAG	This study
mcr-3	F-CTTGCWGAACCAATCCCATTTAC<br>R-TTACCYCGAGCYGTTTCACC<br>P-TAAGCCCACGTTGATGTTYCTGGT	This study
mcr-4	F-GCTGGCTTGGGTATCTAATGAC<br>R-AAGTCCTAGCAAACTGTCGAAC<br>P--ACAGCGAGCAGAACAGGGCGGCTTT	This study
mcr-5	F-GACTCTGTTCATCAGCCTTGTG<br>R-CCCAGTGCAAAGGAGCATTAG<br>P-TCACCCTGCTGTGCAATGGCGTGT	This study
mcr-9	F-TGTTTATCACTGGCTCACTGGT	This study

	R-GGATGGCAGGAAGGATACC	
	P-ACCGGTAAAGACGAACCATATAATAATTGAGC	
sul1	F-CCGTTGGCCTTCCTGTAAAG	[22]
	R-TTGCCGATCGCGTGAAGT	
	P-CAGCGAGCCTTGCGGCGG	
sul2	F-CGGCTGCGCTTCGATT	[22]
	R-CGCGCGCAGAAAGGATT	
	P-MGB-TGCTTCTGTCTGTTTCG	
sul3	F-TAGGCTGCAAAGATAGGGAAAATG	[22]
	R-CAACGCCCACTTCAGTTGTATC	
	P-TGGAGCAGATGTGATTGATTTGGGAGC	
tetA	F-TCTGAGCACTGTCGCGCTC	[22]
	R-ACAGGTGCGCAGGCAAA	
	P-TGGTTCACTCGAACGACGTCACCG	
tetB	F-TGTGGCAGGAAGAATAGCCAC	[22]
	R-ATCCCTGTAAAGCACCTTGCTG	
	P-TTGGCTGGTGGTGGGATCGCTT	
dfrA1	F-TCGAAGAATGGAGTTATCGGGA	[22]
	R-TGTCAGATGTAAAACTTGAACGTGTTA	
	P-TGGGAGCATTACCCAACCGAAAGTATGC	
dfrA12	F-TGCTGCGATGGGAGCC	[22]
	R-GACGGTTCGGTAGAGGCTTG	
	P-TTCGCAGACTCACTGAGGGAAAAGTCGT	
dfrA17	F-GCGTAATCGGTAGTGGTCCTGA	[22]
	R-TTCCGTTCTTTGACACTACTGCAT	
	P-TTGACTCTATGGGTGTTCTTCCAAATCGCA	
IncA/C	F- GAACCAAAGACAAAGACCTGG	This study
	R-TGCTCACGCTTTTGGTCCCAA	
	P-CTCGACGTWATCAAAGACTCACCGCAAATG	
IncFIA	F-ATGGAYCTGTCATGGCGGAA	This study
	R-AACGAACAGATACAGCAKACG	
	P-CGTAAAAATAGCCCGCGRATYGTCCAGT	
IncFIB-AP001918	F-AACTGTTCGGCGAGGTGGA	This study
	R-CAGCTCTTCCGTGGCATCA	
	P-CACTGACACCAAACAATAACAACACCGTAC	
IncFIB-K	F-ACTCGGTGTAATCCAGATAACC	This study
	R-AGCGCAAGGAATCAGCCCA	
	P-ATCGCCTTACGCACCGTGTGGTTC	
IncFIB-	F-TGAACTCAAACAGCTGTCTCTG	This study
pLF82pHCM2	R-GTCCAGGCGYTTGCGTAAC	
	P-AGCCGAAGGGTACGARGACATAAGGATC	
IncFIB-pQIL	F-TCGATGGAGACTGACTTCAAG	This study
	R-CAGACCAGATACAACACCATTC	
	P-CTTCCATTCAGCGAGTTTGCTACATTMTGC	
IncFIB-S	F-CTCCGGTGAAATCGTAAATCTC	This study
	R-GATCTCCACTTTATCAAACCCC	
	P-CTCTGATGCGGCTTGGAGTATTTGTTCC	
IncFII	F-GCACACCATCCTGCACTTAY	This study
	R-CTATGCGGGGAGTATAGTYATG	
	P-CRCAGAAGGAGYGAGCACAGAAAGAAGTC	
	F-TAAGTGTTGGCTCGGTTGCATA	This study

IncHI1A-AF250878	R-TGCTAGCGCCTCCTGAAATC<br>P-TCTACAAACATAAACAAGCGAACTCGCTCA	
IncHI1B-CIT-27	F-GAAAACCGATCTCTTTAAGCTGG<br>R-TTCGAAGCTCAATACGCTGTAG<br>P-ACAGGCCCTGTAYCTGTATCTGGCTGA	This study
IncFIA(HI1)	F-TTGCCCTCAGGAAGCGTTG<br>R-CGCCTCAGTCAGCTCATTTGA<br>P-AGTCTGTCACACTTCACATGGACCTGTC	This study
IncHI1B-MAR	F-TCTTCCCGTCGACACGATAC<br>R-CACACCCATTTCCAGTTCATC<br>P-TCCGGATTTGGCCTGATAATGACCTCATC	This study
IncHI2-1	F-ACCTTGCATCGGTAGTGCTTG<br>R-CGATACAGTTCCCACATAGAAG<br>P-CTCGKAGTGGTCGGTTCACAGTAACG	This study
IncHI2-2	F-CATGATGTGTACCTTACTGGCA<br>R-AACCGGAAGCCGTATTCGC<br>P-CATTGTCCTCCGAGTGTGCGTTCAGC	This study
IncI2	F-GGCTTGAACATCGTTGATCGAT<br>R-CCTGGAAAASACGAGATTACG<br>R-AGGCAAGTTTTCCGCGATTTGTAAATGCAG	This study
IncI-I1	F-GGACGGCAGAATGCGCCAT<br>R-TGGGGGTTTTCCTTTTATGGC<br>P-ATCTTCRGTCTTCTGACTTACTGYCCG	This study
IncK/B/O/Z	F-TGCGCCATAAGGCATTCAGG<br>R-CACAAGCTGTCGCTTATGGC<br>P-CGGTTTCATATATACTTATCCCGTATTCTGTG	This study
IncLM-pOXA48-AF550415	F-AGCGCCAGGAACAGAAGAGT<br>R-GCGAACCCGCTTTTACATAGTA<br>P-CGGAGTTCCAGAGAGAGTACCGGA	This study
IncL/M-pMU407	F-CTGAAGAGTAAACTGACAGACG<br>R-CACCTGCCGCTCAACTACT<br>P-CACAAGCCCTCCGACTACAAACGACTA	This study
IncN-1	F-TTTGAGTTAAGCCGTTCTGCG<br>R-GGATCGTCCGTGGATTTCGA<br>P-ATCGCGGAGCGAGTAGGTGGTGAACT	This study
IncN-2	F-GTTTCTTCACCTTATGGGGATG<br>R-CAGCTGCAAACGACACGCG<br>P-CACAGGGAACCCGTCATAAAACGCTG	This study
IncQ-1	F-GTGCCTGGAAGAACTCAGAG<br>R-AGGGCCAGCATGGATTTACC<br>P-CTAACCACCAACTGCCTGCAAAGGAG	This study
IncR	F-CCAGCCACGGACGTTTAAC<br>R-TGTGGTTATGCCTCATGCAGG<br>P-CCGTTGATTCGCCCAGAGCATTTACC	This study
IncX4-1	F-GACTTCCCTTTCTCGCTTAAC<br>R-TAGAGTTTATCTGGCACATGGC<br>P-TTTAGCAGCCATAGGCAATGTTGACAGCG	This study
IS1294	F-GTCAACTGTCCGCTCAATAATG<br>R-CTTGCCAGACATCAGTAATGGA<br>P-CCGATAACACACCGGCAACAACAAGA	This study
IS26-1	F-TGTTGCAAATAGTCGGTGGTG	This study

	R-CATTCACTCCGCGTTCAGC	
	P-ATTCACTCCGCGTTCAGCCAGCATCT	
IS26-2	F-ATGGAGCTGCACATGAACCC	This study
	R-CTCACGGTAACTGATGCCG	
	P-TTTGCAGTACCAGCGTACGGCCCAC	
IS903	F-TTCAGGCAATACGCACGCTTT	This study
	R-ACAACAGATTACAACCGTCGC	
	P-TTCGTCATTTTGTTCAGCGCTCGTACCA	
IS903B	F-GTTGCATCTGGCCGTTGAC	This study
	R-CCGCAGTTCATCGTGACAGA	
	P-TGCAGACCTGTCGCTGAACAATGTGAC	
IS4321-L	F-CCGGCGTCTGAATGGGATT	This study
	R-AAGCTGGCCAGAATAGCCTG	
	P-CGCCTGATGAACCTCCAGAAAATATACG	
IS4321-R	F-TCTCAGCAGGCAATGCGNG	This study
	R-CATCTTCGTCCAACTTTCGTTTC	
	P-TGCATCGTGTCCGTGAATCCYTGGT	
IS6100	F-TCAGAGGTAGGCTGTCGCT	This study
	R-AGGCCGATCACGGAAAGCT	
	P-CTGCGAAATGGTGGTTGAGCATGCC	
ISAba24	F-CCTCAATCCTTACCTGATGATG	This study
	R-GACTCGTCTTCAATCAGCTGT	
	P-CGGGTTAACCAGCAACTGTCGGAC	
ISAba125	F-TGGTCTCCTCAGCAAATAGCAA	This study
	R-TCTTGAACTTCGGCTGGGC	
	P-TCAACGATACGAGCATTACCCAAGGGTG	
ISApl1	F-GAGTACTTCCTACCGACATCT	This study
	R-CTAGAGCGTGTCGTTTCAGC	
	P-CGCGAGAAATGGTGCTGGAGCTACGT	
ISCR1	F-AGTCAATCGCCCACTCAAACA	This study
	R-GATGACAATCCTCGCAGCG	
	P-CCTCACTCGAAGCCCAAGGTCAAC	
ISCR3/14/27	F-ATGGATGGGCTGCGGATGA	This study
	R-GCTTGTGGCTTTCGTGTGCT	
	P-ATSACCTACCGCATCGCCACCGG	
ISEc33	F-ACGACGCCTGATGCAGAAAG	This study
	R-AACGGATGAACGGGCACAACA	
	P-CCATGCTRATGCTTCATCGGGGTGAAC	
ISEcp1-1	F-GTAGAAGGTCATCAAGAAGGTG	This study
	R-GATCATTTCCGCAGCACCG	
	P-CTAAGAAACTGGGAAACCGATGCTACAATATC	
ISEcp1-2	F-TACCGGATTTGTAAGAAGTGGC	This study
	R-ATCGTCTGATTTGATGTTAGCAAC	
	P-AATACTTACACTGCAAACGGTGCTGCGG	
ISSEn4	F-GCATGAACGATATCGCTGAAC	This study
	R-CATGGTGTTGAGTTGCGCTTA	
	P-CCAGTGCTCATTCAAACATTCATCGCGAA	
Tn2	F-AGATCAGTTGGGTGCACGAG	This study
	R-TTCTGAGAATAGTGTATGCGGC	
	P-ACAGCGGTAAGATCCTTGAGAGTTTTCGC	
ISKpn14	F-GTGGCTTCAGTCTCCGTCT	This study

	R-GAGAAGCGGCATAAGTGAAGG	
	P-CTGTCCCTCCTGTTCCGCTACTGAA	
ISKpn26	F-GAATTTCCGCCACCTGCTC	This study
	R-CCACTGATTGCCTTTCTTGGT	
	P-TGTTCAAGACCATCAATCGCTGGCTGG	
ISKpn19	F-ATACTCAACACCATATCCGGTG	This study
	R-GAGTCGTCATTTCATAGGAGATC	
	P-CTGTTTGGCGTTATTCCGGTATCCGG	
ISKpn27	F-GGAAATCAGGAACTCGACGCT	This study
	R-GGCTGGATTGATGAACTCGC	
	P-ACGTCACCCGCCAAACCATCCGAAA	
intI1	F-GCCTTGATGTTACCCGAGAG	Modified from [27]
	R-GATCGGTCGAATGCGTGT	
	P-CGACGCCCTTGAGCGGAAGTATC	
intI2	F-GACGGCTACCCTCTGTTATCTC	Modified from [27]
	R-TGCACCATACAGCAGCGTA	
	P-TGCGAGTATCCATAACCTGCAAAATGCGTT	
intI3	F-GCCACCACTTGTTTGAGGA	Modified from [27]
	R-GGATGTCTGTGCCTGCTTG	
	P-TGCAGCAAGTGGGTGGCGAATGAGT	

References:

22. Rochegüe, T.; Haenni, M.; Cazeau, G.; Metayer, V.; Madec, J.Y.; Ferry, T.; Lupo, A. An inventory of 44 qPCR assays using hydrolysis probes operating with a unique amplification condition for the detection and quantification of antibiotic resistance genes. *Diagn. Microbiol. Infect. Dis.* **2021**, *100*, 115328. https://doi.org/10.1016/j.diagmicrobio.2021.115328.
23. Quirós, P.; Colomer-Lluch, M.; Martínez-Castillo, A.; Miró, E.; Argente, M.; Jofre, J. Antibiotic-resistance genes in the bacteriophage DNA fraction of human fecal samples. *Appl. Environ. Microbiol.* **2014**, *58*, 606–609. https://doi.org/10.1128/AAC.01684-13.
24. Toner, G.; Russell, C.D.; Hamilton, F.; Templeton, K.; Laurenson, I.F. Phenotypic and molecular detection methods for carbapenemase-producing organisms and their clinical significance at two Scottish tertiary care hospitals. *J. Med. Microbiol.* **2019**, *68*, 560–565. https://doi.org/10.1099/jmm.0.000931.
25. Colomer-Lluch, M.; Jofre, J.; Muniesa, M. Quinolone resistance genes (*qnrA* and *qnrS*) in bacteriophage particles from wastewater samples and the effect of inducing agents on packaged antibiotic resistance genes. *J. Antimicrob. Chemother.* **2014**, *69*, 1265–1274. https://doi.org/10.1093/jac/dkt528.
26. Irrgang, A.; Roschanski, N.; Tenhagen, B.A.; Grobbel, M.; Skladnikiewicz-Ziemer, T.; Thomas, K.; Roesler, U.; Käsbohrer, A. Prevalence of *mcr-1* in *E. coli* from livestock and food in Germany, 2010–2015. *PLoS ONE* **2016**, *11*, e0159863. https://doi.org/10.1371/journal.pone.0159863.
27. Muziasari, W.I.; Pärnänen, K.; Johnson, T.A.; Lyra, C.; Karkman, A.; Stedtfeld, R.D.; Tamminen, M.; Tiedje, J.M.; Virta, M. Aquaculture changes the profile of antibiotic resistance and mobile genetic element associated genes in Baltic Sea sediments. *FEMS Microbiol. Ecol.* **2016**, *92*, fiw052. https://doi.org/10.1093/femsec/fiw052.
