# Supplementary material for: High Throughput Screening of Antimicrobial Resistance Genes in Gram-Negative Seafood Bacteria
Source: Microorganisms. 2022 Jun 15;10(6):1225. doi: 10.3390/microorganisms10061225 (PMC9230514; doi:10.3390/microorganisms10061225)
Supplement: Supplementary file 1 [file microorganisms-10-01225-s001.zip › Table S2.pdf]

Table S2. Controls used to validate the assays.

List of whole genome sequenced strains	List of strains used as positive controls for specific genes (genes)	List of plasmids used as positive controls for specific genes (target)
19383	15F1211 (*mcr-3*)	pCMY-3 (*bla*$_{\text{CMY-3}}$)
26307	E. coli 30 Holland (*bla*$_{\text{TEM-1B}}$)	pFIBpQIL (*IncFIB*)
27838	E. coli 24 Holland (*bla*$_{\text{TEM-1F}}$)	pFIB-pLF82pHCM2 (*IncFIB*)
30457	S. Virchow 58.67 Holland (*bla*$_{\text{TEM-1b}}$, *bla*$_{\text{CTX-M-2}}$, *bla*$_{\text{SHV}}$)	pIncHI1B-Mar (*IncHI1B*)
33777	TZ116 (*bla*$_{\text{VIM-1}}$, *bla*$_{\text{CMY-13}}$)	pIncN-2 (*IncN-2*)
34511	S. concorde05-5343 (*bla*$_{\text{CTX-M-15}}$, *qnrA*)	pISAba24 (*ISAba24*)
34978	59-45 Holland (*bla*$_{\text{CTX-M-3}}$)	pISEc33 (*ISEc33*)
36493	15Q004074 (*mcr-4*)	pISKpn26 (*ISKpn26*)
37007	09CEB904SAL (*bla*$_{\text{CMY-2}}$, *intI1*, *bla*$_{\text{TEM}}$, *armA*, *bla*$_{\text{CTX-M-1group}}$)	pISKpn14 (*ISKpn14*)
38947	Virchow Vagtel 7522438-1 (*bla*$_{\text{CTX-M-9}}$)	pISKpn19 (*ISKpn19*)
41711	PYO pERO (*bla*$_{\text{PER-1}}$)	pISKpn27 (*ISKpn27*)
41726	S. Newport 03-3225 (08CEB3400SAL) (*bla*$_{\text{CMY}}$, *IncA/C2*)	pintI3 (*intI3*)
44091	04CEB8118SAL (*bla*$_{\text{CTX-M-53}}$)	pHI1A (*IncHI1A*)
46558	DAK2 (*bla*$_{\text{SHV-12}}$)	pIncFIB-Mar (*IncFIB*)
46814	Kp37 (*mcr-2*)	pFIBS (*IncFIB*)
46839	RD29 (*mphA*, *bla*$_{\text{TEM}}$, *bla*$_{\text{KPC}}$)	
46877	15F2357 (*mcr-3*)	
46886	KPC-2 (*bla*$_{\text{KPC-2}}$)	
46969	Transkonj (*bla*$_{\text{CMY-1}}$)	
47107	DH10B MSC234 (*bla*$_{\text{OXA-48}}$)	
47303	CTX-M-8 (GIS) (*bla*$_{\text{CTX-M-8}}$)	
47323	E11 (*bla*$_{\text{CTX-M-8}}$)	
47384	E68 (*bla*$_{\text{CTX-M-8}}$)	
10CEB2598SAL	pMUR050 (*aadA2*, *armA*, *aph(3')-Ia*, *sul1*, *intI1*)	
10CEB2956SAL		

10CEB8748SAL<br>12CEB117SAL<br>12CEB2196SAL<br>12CEB3121SAL<br>12CEB432SAL<br>12CEB4337SAL<br>12CEB4367SAL<br>12CEB707SAL<br>1697-O26<br>16-B3PA-006<br>1746-O26<br>1886-O26<br>2D7<br>2K7<br>3383-O103<br>382-O145<br>97HMPL915<br>BEN2200<br>CB12450<br>CB12669<br>CB13483<br>CB13938<br>CB15046<br>CHI-004<br>E5<br>G8<br>L168<br>NV34<br>RDP19<br>RSA-005		

S12LNR3592<br>Z7		
