# Supplementary material for: High Throughput Screening of Antimicrobial Resistance Genes in Gram-Negative Seafood Bacteria
Source: Microorganisms. 2022 Jun 15;10(6):1225. doi: 10.3390/microorganisms10061225 (PMC9230514; doi:10.3390/microorganisms10061225)
Supplement: Supplementary file 1 [file microorganisms-10-01225-s001.zip › Table S3.pdf]

Table S3. Sensitivity and specificity of the assays used in this study. True positive (TP) is the number of positive results for one target obtained by the array on the positive control materials, false positive (FP) is the number of positive results for one target obtained by the array on the control materials which don't carry the target, false negative (FN) is the number of negative results obtained by the array on the control materials which contain the target, true negative (TN) is the number of negative results obtained by the array on control materials which don't carry the target. Sensitivity is the rate of true positive results on the total control materials tested which contain the target (TP/(TP+FN)), specificity is the rate of the true negative results on the total control materials tested which don't carry the target (TN/(TN+FP)).

Target	True positive	False positive	False negative	True negative	**Sensitivity (True positive rate)**	**Specificity (True negative rate)**	Note
aac(6')-Ib-cr	8	0	0	49	**1**	**1**	
aac(3)-IIa	6	1	0	50	**1**	**0.98**	May produce some cross reactions with *aac(3)-IId*
aac(3)-IV	2	0	0	55	**1**	**1**	
ant(2'')-la	4	0	0	53	**1**	**1**	
ant(3'')-Ia	36	2	3	17	**0.92**	**0.89**	May produce some cross-reactions with *ant(2'')-Ia*; Decreased sensitivity due to lack of detection of some *aadA5* variants
aph(3')-la	13	1	1	43	**0.93**	**0.98**	
bla$_{\mathbf{KPC2/3}}$	2	0	0	57	**1**	**1**	
bla$_{\mathbf{NDM1/2}}$	1	0	0	56	**1**	**1**	
bla$_{\mathbf{PER1}}$	1	0	0	57	**1**	**1**	
bla$_{\mathbf{CMY-1}}$	1	0	0	57	**1**	**1**	
bla$_{\mathbf{CMY-2}}$	7	0	0	6	**1**	**1**	These primers/probe were tested on a smaller strain panel
bla$_{\mathbf{CMY-3}}$	8	0	0	54	**1**	**1**	These primers/probe are specific for CMY-2 + CMY-3. They should be used in combination with the primers/probe set CMY-2 to

							determine if CMY-2 or CMY-3 is present.
bla$_{\text{CTX-M9-group}}$	5	0	0	53	**1**	**1**	
bla$_{\text{CTX-M2-group}}$	3	0	0	55	**1**	**1**	
bla$_{\text{CTX-M1-group}}$	25	0	0	36	**1**	**1**	
bla$_{\text{CTX-M8-group}}$	3	0	0	57	**1**	**1**	
dfrA1	19	0	0	38	**1**	**1**	
dfrA12	5	0	0	52	**1**	**1**	
dfrA17	4	0	0	53	**1**	**1**	
floR	6	2	1	48	**0.86**	**0.96**	
***mcr*-1**	6	0	0	51	**1**	**1**	
***mcr*-2**	1	0	0	57	**1**	**1**	
***mcr*-3**	3	0	0	56	**1**	**1**	
***mcr*-4**	1	0	0	57	**1**	**1**	
***mcr*-5**	1	0	0	56	**1**	**1**	
***mcr*-9**	3	0	0	54	**1**	**1**	
bla$_{\text{OXA-48}}$	2	0	0	56	**1**	**1**	
qnrB1	2	0	0	55	**1**	**1**	
qnrS	3	0	0	54	**1**	**1**	
bla$_{\text{SHV}}$	9	0	0	50	**1**	**1**	
strA	31	0	0	26	**1**	**1**	
strB	31	0	0	26	**1**	**1**	
sul1	32	0	0	26	**1**	**1**	
sul2	34	0	0	23	**1**	**1**	
sul3	8	0	1	48	**0.89**	**1**	
bla$_{\text{TEM}}$	43	0	0	24	**1**	**1**	
tetA	28	0	0	29	**1**	**1**	

tetB	12	0	0	45	**1**	**1**	
**IncA/C**	4	0	0	54	**1**	**1**	
**IncFIA**	11	0	0	46	**1**	**1**	
**IncFIB**	28	0	6	25	**0.82**	**1**	
**IncFII**	26	0	5	26	**0.84**	**1**	
**IncHI1A**	2	0	0	56	**1**	**1**	
**IncHI1B**	3	0	1	54	**0.75**	**1**	
**IncHI2**	10	0	0	47	**1**	**1**	
**IncI2**	3	0	0	54	**1**	**1**	
**IncI-I1**	15	0	1	41	**0.94**	**1**	
**IncK/B/O/Z**	9	0	0	48	**1**	**1**	
**IncL/M**	2	0	0	55	**1**	**1**	
**IncN-1**	1	0	0	57	**1**	**1**	
**IncN-2**	1	0	0	57	**1**	**1**	
**IncQ-1**	14	0	0	43	**1**	**1**	
**IncR**	4	0	0	53	**1**	**1**	
**IncX4**	9	0	0	49	**1**	**1**	Specific for IncX4_1, does not include IncX4_2
**IS*SEn4***	7	1	0	49	**1**	**0.98**	
**IS*Kpn14***	1	0	0	57	**1**	**1**	Only exact IS*Kpn14* sequences are targeted
**IS*Kpn19***	4	0	0	54	**1**	**1**	
**IS*Kpn26***	23	1	1	33	**0.96**	**0.97**	
**IS26**	42	8	0	8	**1**	**0.5**	
**IS*4321***	20	0	0	37	**1**	**1**	Targets both IS4321-L and IS4321-R
**IS*Kpn27***	1	0	0	57	**1**	**1**	
**IS*903* + IS*903B***	21	0	2	34	**0.91**	**1**	

**ISCR3/14/27**	2	0	0	55	**1**	**1**	
**ISCR1**	9	0	0	48	**1**	**1**	
**IS*Ec33***	1	0	0	57	**1**	**1**	Only exact IS*Ec33* sequences are targeted
**IS*Aba24***	2	0	0	56	**1**	**1**	
**IS*Aba125***	1	0	0	56	**1**	**1**	
**IS*Apl1***	2	0	0	55	**1**	**1**	
**IS*Ecp1***	27	0	0	30	**1**	**1**	
**IS*1294***	1	0	0	56	**1**	**1**	
**IS*6100***	14	0	0	43	**1**	**1**	
intI1	41	2	2	14	**0.95**	**0.88**	
intI2	9	0	0	48	**1**	**1**	
intI3	1	0	0	57	**1**	**1**	
**Tn2**	32	0	1	24	**0.97**	**1**	
